# IL-12 and IL-27 regulate the phagolysosomal pathway in mycobacteria-infected human macrophages

**DOI:** 10.1186/1478-811X-12-16

**Published:** 2014-03-11

**Authors:** Joo-Yong Jung, Cory M Robinson

**Affiliations:** 1 Department of Pathology, Microbiology, and Immunology, University of South Carolina School of Medicine, 6439 Garners Ferry Road, 29209 Columbia, SC, USA

**Keywords:** BCG, V-ATPase, CD63, Cathepsin D, Phagolysosome, Human macrophages

## Abstract

**Background:**

The cytokine environment at the site of infection is important to the control of mycobacteria by host macrophages. During chronic infection immunosuppressive cytokines are likely to favor mycobacterial growth, persistence, and an avoidance of proper antigen processing and presentation. The activity of interleukin (IL)-27 toward macrophages is anti-inflammatory and this compromises control of mycobacteria. Modulation of the cytokine environment may enhance both protective and vaccine-induced responses.

**Results:**

In this study we showed that supplying IL-12 and neutralizing IL-27 enhanced acidification and fusion of mycobacterial-containing phagosomes with lysosomes. This was achieved by phagosomal acquisition of vacuolar ATPase (V-ATPase) and CD63. Both V-ATPase and CD63 protein levels were increased by the addition of IL-12 and neutralization of IL-27. In addition, cathepsin D associated with the bacteria and matured to the active form when IL-12 was supplied and IL-27 was neutralized. Lysosomal acidification and cathepsin D activity were associated with control of mycobacteria. The acidification of lysosomes, association with mycobacteria, and maturation of cathepsin D required macrophage production of IFN-γ and signaling through signal transducer and activator of transcription (STAT)-1. In contrast, STAT-3 signaling opposed these events.

**Conclusions:**

Our results have identified novel influences of IL-12, IL-27, and STAT-3 on lysosomal activity and further demonstrate that modulating the cytokine environment promotes enhanced trafficking of mycobacteria to lysosomes in human macrophages. This has important implications in approaches to control infection and improve vaccination. Overcoming bacterial resistance to lysosomal fusion may expand the repertoire of antigens presented to the adaptive arm of the immune response.

## Background

*Mycobacterium tuberculosis* (MTB) is an intracellular human pathogen responsible for an enormous burden of human disease. In 2011, there were approximately 8.7 million incident cases of tuberculosis (TB) globally and nearly a third of the world population has been infected [1]. The only currently available vaccine for TB is *Mycobacterium bovis* bacille Calmette-Guerin (BCG). BCG effectively protects against disseminated tuberculosis, such as miliary TB and tuberculous meningitis in children [2,3]. However, the BCG vaccine has not been consistently effective at preventing pulmonary tuberculosis, and thus the effects of BCG on combating the global burden of tuberculosis have been limited.

Several hypotheses have been proposed to explain the limitations of BCG vaccination. These include interference by environmental mycobacteria, genetic differences in the human population, and differences between BCG substrains [4]. Recently, it has been proposed that mycobacterial antioxidants, such as iron-cofactored superoxide mutase [5], and secA2 secretion suppress host immunity [6], resulting in reduction of vaccine efficacy. Suppression of host immunity could be mediated by anti-inflammatory cytokines. BCG-infected mice express high levels of the Th2 cytokines interleukin (IL)-5 and IL-13 [7]. Similarly, IL-4 and TGF-β are known to be increased in tuberculosis patients [8]. Thus, another possible explanation for limited effectiveness of BCG may be inhibition of host immunity through the action of immune suppressive cytokines.

IL-27 is produced by antigen presenting cells in response to a variety of activation stimuli, notably microbial-derived products [9]. IL-27 activates Janus kinases (JAK) and signal transducer and activator of transcription (STAT)-1 and STAT-3 through its receptor composed of WSX-1 and gp130 [10]. IL-27 was originally described as a soluble factor that promotes Th1 activity [11]. STAT-1 and STAT-3 modulate the T-cell specific transcription factors such as T-bet (Th1) or GATA-3 (Th2) [12]. However, IL-27 also negatively regulates Th1 cells, highlighting its paradoxical nature [13]. Similarly, IL-27 inhibits differentiation of Th17 cells and production of IL-17 by inducing IL-10 producing Tr-1 cells through STAT-1 and STAT-3 [14]. Immunosuppressive activity of IL-27 has been described toward a number of immune cell types involved in innate immune responses [10]. IL-27 induces an immunosuppressive phenotype in murine DCs by increasing expression of B7-H1 in a STAT-3-dependent manner [15,16]. Proinflammatory cytokine production is inhibited in both human and murine macrophages by IL-27 [17,18].

IL-27 produced by human macrophages during MTB infection opposes inflammatory responses [17,19-21]. Treatment with IL-12 in conjunction with neutralization of IL-27 restricts the growth of MTB and requires the proinflammatory mediators IFN-γ, TNF-α, and IL-18 [17,19]. This immunomodulation promotes more effective macrophage-mediated immunity. Even though immunological parameters involved with the treatment of IL-12 and sIL-27R that improve mycobacterial control have been revealed [19], the intracellular mechanisms involved have not been elucidated.

MTB arrest phagosomes at an early stage of endosomes by blocking phagosomal maturation [22]. This prevents fusion with late endosomes and lysosomes. Beyond the implications in host-mediated control of mycobacteria, this limits antigen presenting cell processing of mycobacterial antigen. *Mycobacterium bovis* BCG also avoid phagosomal fusion with lysosomes as efficiently as MTB [23,24]. In doing so, BCG may limit the range of antigens that are processed for presentation by major histocompatibility complex (MHC) class II. The phagosomal/lysosomal trafficking pathway involves a variety of host molecules including proteins and phospholipids. Phagosomal maturation occurs through the acquisition of several lysosomal markers, such as lysosome associated membrane protein LAMP-1, LAMP-2, and CD63. In the final stage of maturation, the phagosome acquires V-ATPase and cathepsins in a syntaxin6-dependent manner [25].

The proinflammatory cytokine IFN-γ promotes phagosomal maturation by inducing acidification of phagosomes [26]. IFN-γ treatment in murine macrophages leads to acidification of mycobacterial-containing phagosomes [23]. Additionally, IFN-γ treatment of monocyte-derived macrophages increased lysosomal fusion with endosomes [26]. However, there is some evidence that anti-inflammatory cytokines can inhibit the phagosomal/lysosomal pathway [21,23,26]. MTB-infected macrophages from IL-10 knock-out mice exhibit an increased level of acidification [23]. IL-10 decreased fusion of horse radish peroxidase (HRP) containing phagosomes with lysosomes [26]. Recently we have demonstrated that IL-27 decreases phagosomal acidification through inhibition of V-ATPases [21].

Based on these reports, treatment of infected macrophages with IL-12 and sIL-27R could promote an environment that is unfavorable for mycobacterial growth by enhancing phagosomal maturation and fusion with lysosomes. The objective of this work was to evaluate the influence of IL-12 and IL-27 on the phagosomal/lysosomal pathway during infection by BCG. Promoting enhanced delivery of BCG to lysosomes may enhance the magnitude and diversity of antigen presentation. Treatment of IL-12 combined with neutralization of IL-27 increased the expression of CD63 and V-ATPase. The consequence was enhanced phagosomal acidification and localization of cathepsinD at the BCG-containing phagosome. This immunomodulatory approach that overcomes phagolysosomal resistance may not only be important for controlling bacterial growth but also increasing the repertoire of BCG antigens that are presented during vaccination and improve the efficacy of BCG.

## Results

### BCG infection of human macrophages increases the production of IL-27

**Figure 1:**
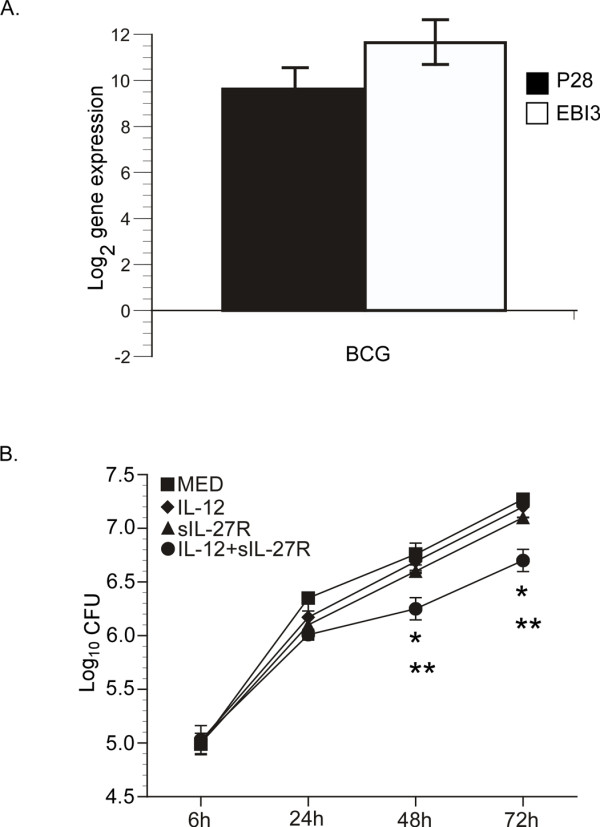
**IL-27 expression during infection promotes mycobacterial growth. (A)** Macrophages were infected with BCG for 48 h. Quantitative analysis of p28 and EBI3 is presented as the mean log_2_ change in gene expression of duplicate samples ± standard error for a representative experiment of three. Values were normalized to the mean expression of GAPDH within a sample group and expressed relative to that of medium alone. **(B)** Macrophages restrict the intracellular growth of BCG. Macrophages were treated with IL-12, sIL-27R, or their combination for 6 h prior to BCG infection for an additional 6, 24, 48, or 72 h. Data is represented as the mean CFUs recovered from infected macrophages ± standard error for two combined experiments. A student’s *t* test was used to establish statistical significance in the 95% confidence interval between individual sample groups as indicated; *indicates that IL-12 and sIL-27R is significantly different from medium alone (MED). **indicates that IL-12 and sIL-27R is significantly different from all other treatments.

We previously demonstrated that MTB infection induced IL-27 gene expression in human macrophages [17]. This is also true in response to BCG. Human macrophages were infected with BCG (MOI 10) for 48 h and IL-27 transcripts were analyzed by real time PCR. Transcript levels for both p28 and EBI3 were markedly increased by BCG infection (Figure 1A). Additionally, human macrophages express the IL-27 receptor, composed of WSX-1 and gp130 [17]. Thus, human macrophages can respond to IL-27 and do so with implications in the control of mycobacteria [17,19]. Neutralization of IL-27 along with supplying IL-12 restricts MTB growth by 24 h and this becomes more pronounced through 72 h [17]. Similar results were observed in BCG-infected macrophages. Macrophages were infected with BCG following treatment with IL-12, sIL-27R, or their combination for 6 h. BCG were then enumerated at 24, 48, and 72 h. The combination of IL-12 and sIL-27R was most effective at reducing the bacterial burden relative to other treatments (Figure 1B). Log_10_ reductions of 0.51 and 0.53 colony forming units (CFUs) were observed at 48 and 72 h, respectively (Figure 1B). These results were not influenced by a reduction in macrophage viability; there was no change in toxicity relative to uninfected macrophages with any treatment condition (Additional file 1: Figure S1). These data demonstrate that treatment with IL-12 and sIL-27R decreased the number of viable BCG comparable to MTB [17].

### Supplying IL-12 and neutralizing IL-27 induced lysosomal acidification

**Figure 2:**
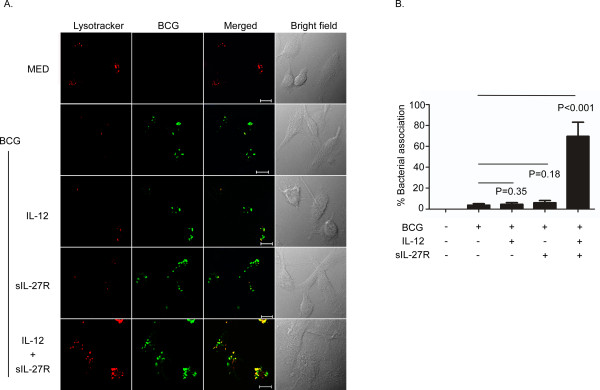
**Treatment with IL-12 and sIL-27R induced phagosomal acidification in BCG-infected macrophages.** Macrophages were treated with IL-12, sIL-27R, or their combination for 6 h prior to infection with SYTO-9® -stained BCG for 48 h. Lysotracker (100 nM) was added during the last hour of infection. **(A)** The images shown are from an individual experiment representative of three independent experiments (scale bar = 10 μm). **(B)** The percent association was calculated based on Pearson’s coefficient as described in the Methods section. These data are the combined results from three independent experiments ± standard error. A student’s *t* test was used to establish statistical significance in the 95% confidence interval between individual sample groups as indicated.

MTB and BCG arrest phagosomal maturation; this is characterized by inhibition of luminal acidification and the absence of mature lysosomal hydrolases [22]. Increased localization of mycobacteria with lysosomes in IL-10-deficient murine macrophages has been reported [23]. More recently we have shown that exogenously supplied IL-27 inhibits latex bead-containing phagosomal acidification [21]. Thus, changing the cytokine environment in macrophage cultures may reverse mycobacterial phagosome arrest and account for the decrease in intracellular growth (Figure 1). To address this possibility, human macrophages were treated with medium alone, IL-12 (5 ng/ml), sIL27R (10 μg/ml) or their combination for 6 h prior to infection with BCG for 48 h. No individual or combination of reagents influenced lysosomal signal in the absence of infection (Additional file 2: Figure S2). The combination of IL-12 and sIL-27R during infection promoted a profound increase in lysotracker signal (red) indicative of increased lysosomal acidification. This change was so profound relative to any other treatment that we were unable to discern the subcellular compartmentalization of lysosomes (Additional file 3: Figure S3). Therefore, to address association of lysosomes with BCG, we reduced the signal intensity of the red channel to a level that allowed us to observe discreet lysosomes in BCG-infected macrophages treated with IL-12 and sIL-27R. The same settings were then applied to all other treatment conditions. Low-intensity acidified lysosomes were observed during BCG infection in the absence of additional treatment (Figure 2). Additional treatment with IL-12 or sIL-27R individually did not significantly increase lysosomal acidification. The increased frequency and magnitude of acidified compartments observed in response to the combination of IL-12 and sIL27R were predominantly associated with SYTO-9®-labeled BCG (Figure 2A and B). This enhancement of association by IL-12 and sIL-27R is likely due in part to the increase in lysosomal acidification (Additional file 3: Figure S3). Collectively, these results suggest that supplying IL-12 and neutralizing IL-27 enhances the formation of acidified lysosomes and these lysosomes are associated to BCG-containing phagosomes.

### Supplying IL-12 and neutralizing IL-27 induced the expression of CD63 and enhanced association with BCG

**Figure 3:**
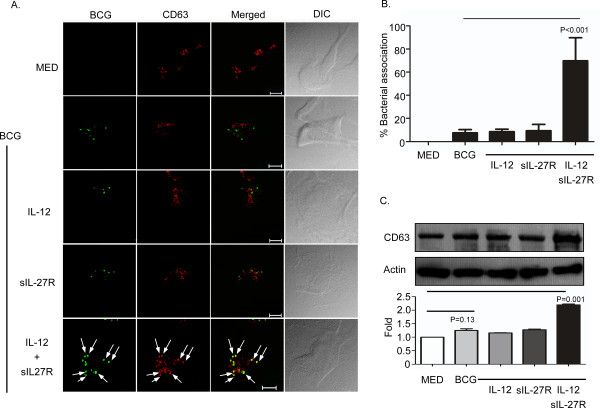
**Treatment of BCG-infected macrophages with IL-12 and sIL-27R increases expression of CD63.** Macrophages were treated with IL-12, sIL-27R, or their combination for 6 h and then infected with SYTO-9® -stained BCG for 48 h. **(A)** The cultures were subsequently fixed with 4% PFA, permeabilized, and labeled with anti-CD63 antibody (red). Representative images from three experiments are shown (scale bar = 10 μm). Arrows indicate the association of CD63 with BCG. **(B)** Percent bacterial association was calculated as described in the Methods section. These data are representative results from three independent experiments. **(C)** Cells were lysed to collect whole-cell lysates for immunoblot analysis (upper panel). CD63 or actin was labeled as described in the Methods Section. An image representative of two experiments is shown. The ratio of CD63/actin band intensity was expressed relative to medium alone (lower panel). Data plotted here are the result of two combined experiments. **(B, C)** A student’s *t* test was used to establish statistical significance in the 95% confidence interval between individual sample groups as indicated.

Since IL-12 and sIL-27R enhanced phagolysosomes (Figure 2), we wanted to address whether this treatment led to association of mycobacteria with markers of mature lysosomes. CD63, also known as lysosomal integrated membrane protein-1 (LIMP-1), localizes to the lysosomal compartment and is known to be excluded from mycobacteria-containing phagosomes [27]. To address this, macrophages were treated and infected as described earlier and then immunolabeled for CD63. Similar to the description above, there was such an overwhelming increase in the amount of CD63 signal (red) detected during treatment with IL-12 and sIL-27R (Additional file 4: Figure S4), we could not discern the subcellular compartment of CD63-containing vacuoles. Therefore, we reduced the signal intensity of the red channel in this treatment condition such that we could discern discreet vacuoles that contained CD63 and applied the same settings to all treatment conditions. The combination of IL-12 and sIL-27R significantly enhanced expression of CD63 as well as its association with BCG as compared to all the other treatments (Figure 3A and B). This may in part be a reflection of increased CD63 protein expression. The increased expression levels were confirmed by immunoblot analysis of CD63 in whole-cell lysates (Figure 3C). An analysis of band intensity from two immunoblots done with independent blood donors showed an approximate two-fold increase in CD63 expression (Figure 3C, lower panel).

### Supplying IL-12 and neutralizing IL-27 induced the expression of vacuolar ATPases

**Figure 4:**
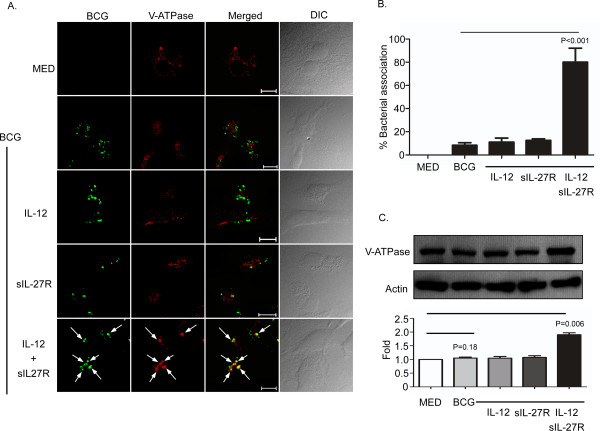
**Treatment of BCG-infected macrophages with IL-12 and sIL-27R increases expression of V-ATPase.** Macrophages were treated with IL-12, sIL-27R, or their combination for 6 h and then infected with SYTO-9®-stained BCG for 48 h. **(A)** The cultures were subsequently fixed with 4% PFA, permeabilized, and stained with anti-VATPase H antibody (red). Representative images from three experiments are shown. Arrows indicate the association of V-ATPase with BCG. **(B)** The percent bacterial association was calculated as described in the Methods section. These data are representative results from three independent experiments. **(C)** Macrophage cultures were lysed to collect whole-cell lysates for immunoblot analysis (upper panel). V-ATPase or actin was labeled as described in the Methods section. An image representative of two experiments is shown. The ratio of V-ATPse/actin band intensity was expressed relative to medium alone (lower panel). Graphical data plotted are the result of two combined experiments. **(B, C)** A student’s *t* test was used to establish statistical significance in the 95% confidence interval between individual sample groups as indicated.

Intracellular V-ATPases are multisubunit enzymes that when localized to the phagosome allow for a decrease in pH from 6.5 to 5.0 [28]. This acidic environment inhibits the growth of microorganisms and further enhances the recruitment and activities of hydrolytic enzymes [29]. Mycobacterial-containing phagosomes exclude host V-ATPases to inhibit phagosomal acidification [22,30,31]. Moreover, IL-27 decreases the expression of V-ATPases and association with latex bead-containing phagosomes [21]. Since supplying IL-12 and neutralizing IL-27 increases phagosomal acidification, we hypothesized that this change in cytokine environment may enhance the expression or localization of V-ATPases. To address this hypothesis, macrophages were treated as described earlier, infected, and then immunolabeled for V-ATPases. Similar to other lysosomal markers, the V-ATPase signal (red) was drastically increased by treatment with IL-12 and sIL-27R (Additional file 4: Figure S4). To further study the subcellular compartment of V-ATPase, we reduced the signal intensity of the red channel in this treatment condition such that we could discern discreet V-ATPase vacuoles and applied the same settings to all other treatment conditions observed. In addition to increasing the expression level, treatment with IL-12 and sIL-27R increased the association of V-ATPases with BCG relative to all other treatments (Figure 4A and B). The increase in protein expression during treatment with IL-12 and sIL-27R was further confirmed by immunoblot analysis of V-ATPase (Figure 4C). Analysis of the band intensity from immunoblots that corresponded to two independent blood donors showed an approximate two-fold increase of V-ATPase expression (Figure 4C lower panel).

### Supplying IL-12 and neutralizing IL-27 induced the formation of mature cathepsin D and enhanced association with BCG

**Figure 5:**
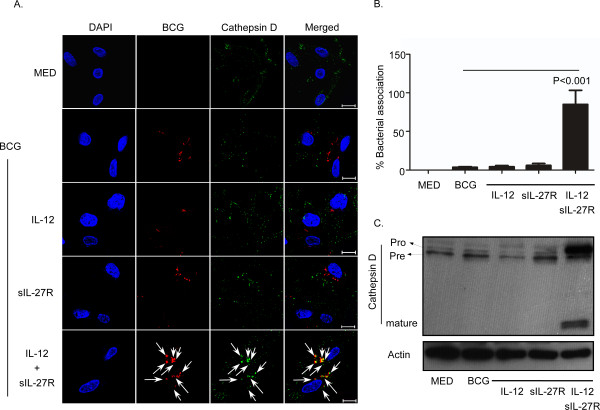
**Treatment with IL-12 and sIL-27R increases association and maturation of cathepsin D during mycobacterial infection.** Macrophages were treated with IL-12, sIL-27R, or their combination for 6 h and then infected with SYTO-61® -stained BCG for 48 h. **(A)** Samples were subsequently fixed with 4% PFA, permeabilized, and stained with anti-cathepsin D antibody (green). Representative images from three experiments are shown. Arrows indicate association between BCG (red) and cathepsin D (green). **(B)** The percent association was calculated as described in the Methods section. These data are representative results from three independent experiments. A student’s *t* test was used to establish statistical significance in the 95% confidence interval between individual sample groups as indicated. **(C)** Macrophage cultures were lysed to collect whole-cell lysates for immunoblot analysis. Cathepsin D or actin was labeled as described in the Methods section. Pro; procathepsin D (52 kDa), Pre; Precathepsin D (48 kDa), mature; mature cathepsin D (approximately 24 kDa). An image representative of two experiments is shown.

Another molecule that is important for the destruction of intracellular bacteria and antigen processing is cathepsin D. Cathepsin D is a soluble lysosomal endopeptidase synthesized in the endoplasmic reticulum as pre-procathepsin D. Following removal of the signal peptide, the 52 kDa procathepsin D is localized to endosomes, phagosomes, or lysosomes [29]. The acidification of phagosomes leads to cleavage of a 44 amino acid N-terminal propeptide that results in a 48 kDa single chain active enzyme. This form can undergo additional cleavage to yield a mature and active lysosomal protease (24 to 30 kDa) [29]. Cathepsin D is involved in bacterial antigen processing by breaking down bacteria present in the phagolysosome [32]. In a murine macrophage model, *M. avium*-containing phagosomes acquired the preform of cathepsin D but this protein was not processed to the cleaved active form due to the blockage of pH reduction in mycobacterial phagosomes [33]. Thus, there was an absence of mycobacterial degradation in the phagosome. We investigated whether cathepsin D was processed to the mature form in acidified BCG-containing phagosomes. To address this, we utilized an antibody that recognizes only the active form (24 to 30 kDa) of cathepsin D. Treatment with IL-12 and sIL-27R enhanced association of active cathepsin D with BCG phagosomes by approximately 85%, as compared with 3.6% during infection alone (Figure 5A and B). To further confirm cathepsin D processing and the relationship to total protein levels, we performed immunoblot analysis with an antibody that detects all three forms of cathepsin D. Immunoblot results demonstrated that treatment with IL-12 and sIL-27R led to processing of procathepsin D (Figure 5C Pro; 52 kDa on top band) to an active enzyme form (Figure 5C pre; 48 kDa on second band from the top) that was further cleaved to yield a fully mature and active form (Figure 5C Mature; ≤30 kDa bottom band). However, this process did not occur in the absence of IL-12 and sIL-27R (Figure 5C lanes 1 and 2). These data confirm the acidification of BCG phagosomes in macrophages stimulated with IL-12 and sIL-27R, and further demonstrate the functional proteolytic consequence.

### Enhanced fusion of mycobacterial phagosomes with lysosomes restricts BCG growth

**Figure 6:**
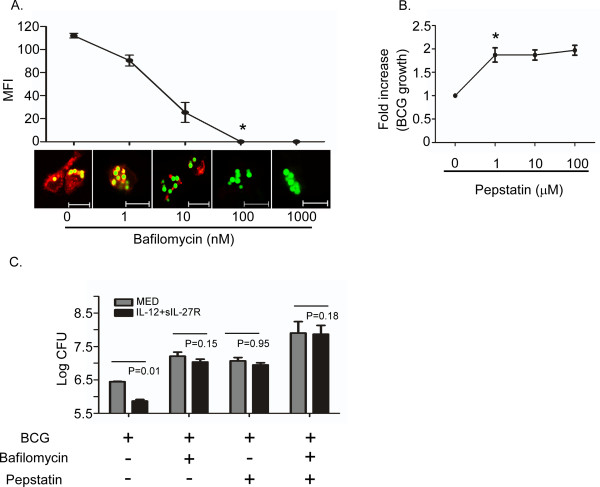
**Pharmacological inhibition of V-ATPase and cathepsin D reversed the restriction of BCG growth in IL-12 and sIL-27R-treated macrophages. (A)** Macrophages were treated with bafilomycin (0–1000 nM) for 4 h and then subsequently treated with green fluorescent labeled latex beads for additional 48 h. Acidified lysosomes (red) were stained with LysoTracker (100 nM). The images shown are typical results from three separate experiments. MFI was calculated as described in the Methods section. A student’s *t* test was used to establish statistical significance in the 95% confidence interval; * indicates P < 0.001 compared to the untreated sample. **(B)** Pepstatin promotes the growth of BCG in macrophages in a dose-dependent manner. Macrophages were treated with pepstatin (0–100 μM) for 4 h prior to infection and then infected with BCG for an additional 48 h. BCG were stained with Auromine O and fluorescence was measured. The mean fluorescent intensity (MFI) ± standard error for two independent experiments is shown. A student’s *t* test was used to establish statistical significance in the 95% confidence interval; * indicates P < 0.001 compared to the untreated sample. **(C)** Treatment of bafilomycin and/or pepstatin reversed growth restriction of BCG by IL-12 + sIL-27R-treated macrophages. Macrophages were either untreated or treated with IL-12 + sIL-27R, bafilomycin, and/or pepstatin for 6 h and then subsequently infected with BCG for 48 h. Data is represented as the mean CFUs recovered from infected macrophages ± standard error at 48 h for three independent experiments. A student’s *t* test was used to establish statistical significance in the 95% confidence interval between individual sample groups as indicated.

Supplying IL-12 and neutralizing IL-27 promoted an intracellular environment that reduced mycobacterial growth (Figure 1). This was consistent with increased CD63 and V-ATPase expression that led to acidification of the BCG-containing phagosome, further evident by maturation of cathepsin D (Figures 2, 3*,*4, 5). To confirm that the acidification of BCG-containing phagosomes was responsible for the control of mycobacterial growth when supplying IL-12 and neutralizing IL-27, we made use of pharmacological inhibitors that block acidification. Bafilomycin is an antibiotic isolated from *Streptomyces griseus* that is a specific inhibitor of V-ATPase [34]. Importantly, it does not have an effect on other proton pump ATPases such as P-type and F-type ATPases [35]. The effective bafilomycin concentration that reduced lysosomal acidification was determined by treatment with various concentrations (0–1000 nM) and examination of fluorescein-conjugated latex bead association with lysosomes (Figure 6A). A dose-responsive reduction in lysotracker staining was observed, and 100 nM bafilomycin completely inhibited phagosomal acidification (Figure 6A). Pepstatin A is a selective inhibitor of acidic (aspartic) proteases that forms a 1:1 complex with cathepsin D [36]. Auramine O staining of BCG was increased approximately two-fold in the presence of 1 μM pepstatin (Figure 6B). There was no further influence on growth with higher concentrations of pepstatin, indicating a maximal effect (Figure 6B). From these results, we chose 100 nM of bafilomycin and 1 μM of pepstatin A for inhibition of V-ATPase and cathepsin D, respectively, in subsequent experiments. These concentrations of bafilomycin and pepstatin were not toxic to human macrophages (Additional file 5: Figure S5). Treatment with bafilomycin and pepstatin reverses the reduction of BCG growth mediated by IL-12 and sIL-27R (Figure 6C). There was also an expected effect of inhibitors on infected cultures in medium alone, but importantly, there was no control of mycobacterial growth mediated by IL-12 and sIL-27R (Figure 6C). This suggests that supplying IL-12 and neutralizing IL-27 specifically enhances trafficking to lysosomes to control mycobacterial growth.

### IFN-γ is important for the enhancement of phagosomal acidification when IL-12 is supplied and IL-27 is neutralized

**Figure 7:**
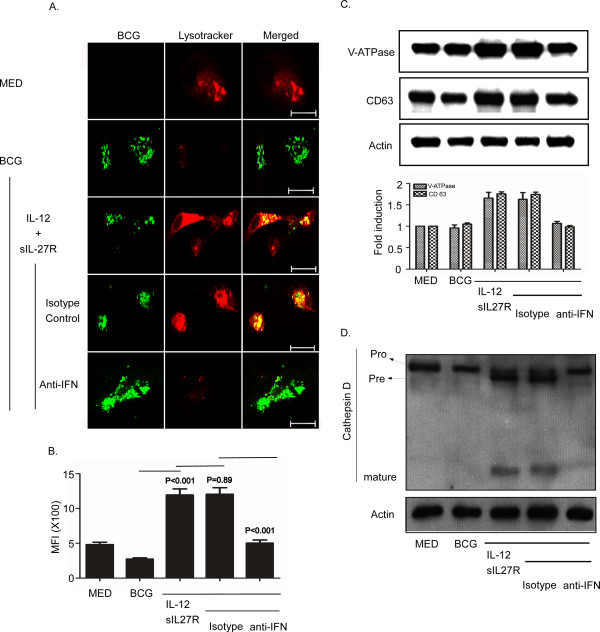
**IFN-γ is required for phagosomal acidification during treatment with IL-12 and sIL-27R.** Macrophages were treated with IL-12 and sIL-27R in the presence or absence of isotype control or IFN-γ neutralizing antibodies for 6 h prior to infection with SYTO-9®-stained BCG for 48 h. **(A, B)** Lysotracker (100 nM) was added in the last hour of infection. Samples were subsequently fixed with 4% PFA. **(A)** The images shown are representative of typical results in three experiments. **(B)** The MFI was analyzed as described in the Methods section and represents data from three combined experiments. A student’s *t* test was used to establish statistical significance in the 95% confidence interval between individual sample groups as indicated. **(C, D)** Cells were lysed to collect whole-cell lysates for immunoblot analysis (upper panel). **(C)** V-ATPase, CD63, or actin was labeled as described in the Methods section. An image representative of three experiments is shown. The ratio of proteins/actin band intensity was expressed relative to medium alone (lower panel). Data plotted here are the result of two combined experiments. **(D)** Cathepsin D or actin was labeled as described in the Methods section. Pro; procathepsin D (52 kDa), Pre; Precathepsin D (48 kDa), mature; mature cathepsin D (approximately 24 kDa). An image representative of two experiments is shown.

IFN-γ is also known to enhance phagosomal acidification [26]. As such, we hypothesized that neutralization of IFN-γ may reverse the enhancement of phagosomal acidification and subsequent effect on cathepsin D maturation. Human macrophages were treated with IL-12 and sIL-27R for 6 h prior to infection. Isotype control or neutralizing antibodies for IFN-γ were also included and followed by infection with BCG. Neutralization of IFN-γ completely blocked the increased phagosomal acidification mediated by IL-12 and sIL-27R (Figure 7A). The MFI was significantly decreased to the level observed in untreated and uninfected macrophages (Figure 7B). This block in acidification was consistent with the decreased protein expression of V-ATPase and CD63 that was comparable to that of untreated and uninfected macrophages (Figure 7C). Since processing of cathepsin D to the mature form requires acidification, we evaluated the effect of blocking IFN-γ by immunoblot analysis of cathepsin D. Neutralization of IFN-γ during IL-12 and sIL-27R treatment blocked the maturation of cathepsin D (Figure 7D, lane 5).

### STAT-1 is an important regulator of lysosomal changes mediated by supplying IL-12 and neutralizing IL-27

**Figure 8:**
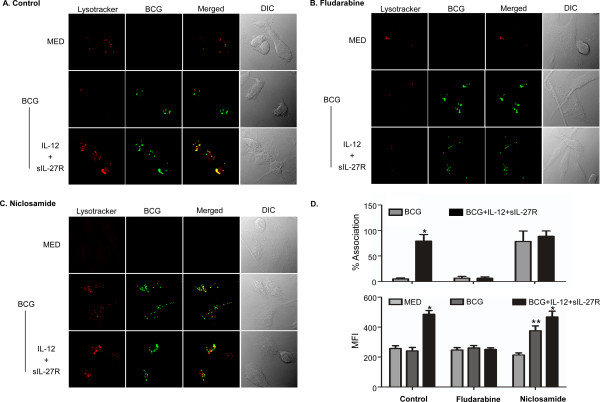
**Inhibition of STAT-1 prevented the increase in phagosomal acidification during treatment with IL-12 and sIL-27R.** Human macrophages were either treated with **(A)** DMSO as a control, **(B)** fludarabine, or **(C)** niclosamide for 6 h and then infected with BCG for an additional 48 h. Lysotracker was added at the end of infection for 1 h. **(A, B, C)** The images shown are representative of two independent experiments. **(D)** The percent association and MFI were calculated as described in the Methods section. These data are the combined results of two independent experiments ± standard error. A student’s *t* test was used to establish statistical significance in the 95% confidence interval between individual sample groups as indicated; *indicates that IL-12 and sIL-27R is significantly different from BCG alone (BCG). **indicates that BCG alone (BCG) is significantly different from MED alone (MED).

IL-27 binding of its receptor induces phosphorylation of STAT-1 and STAT-3 [10]. We have also demonstrated the involvement of IFN-γ that signals to induce phosphorylation of STAT-1 [19]. Therefore, to dissect the signaling molecules that may direct the cellular responses involved with the influences on the lysosomal pathway reported here, we made use of chemical inhibitors selective for STAT1 (fludarabine, [37]) or STAT3 (niclosamide, [38]). Human macrophages were treated with or without IL-12 and sIL-27R for 6 h in the presence or absence of fludarabine (250 μM) or niclosamide (4 μM) and then infected with BCG as before for an additional 48 h. STAT-1 inhibition had no influence during infection alone, but it completely prevented the increase in acidification and association with BCG mediated by IL-12 and sIL-27R compared to the control group (no inhibitor, Figure 8A, B, and D). In contrast, inhibition of STAT-3 during BCG infection alone led to an increase in lysosomal acidification and corresponding association with BCG, albeit the latter to a greater extent (Figure 8A, C, and D). During treatment with IL-12 and sIL-27R, STAT-3 inhibition did not alter the acidification of lysosomes or their association with BCG relative to the control (no inhibitor, Figure 8A, C, and D). This is consistent with limited STAT-3 signaling as a result of IL-27 neutralization. Collectively, these results demonstrate that signaling through STAT-1 positively regulates lysosomal activity during mycobacterial infection while STAT-3 opposes this pathway.

## Discussion

Effective innate immunity involves the activation of macrophages. Activated macrophages utilize a variety of molecules and intracellular pathways to combat microbial pathogens. Among these is the maturation of phagosomes to lysosomes that is accompanied by a decrease in pH. Mycobacteria evolved to avoid this pathway by excluding the effector proteins involved in phagosomal acidification and lysosomal fusion (CD63, V-ATPase, and cathepsin D) from their vacuoles [22]. BCG has been safely used for nearly a century, but the protective efficacy against TB is highly variable [8]. BCG is highly similar to MTB in antigenic composition [39,40] and equally efficient at avoiding phagosomal maturation and lysosomal fusion [23,24]. Consequently, only a limited repertoire of BCG antigens may be presented in the initiation of an adaptive response. This may contribute to the limited vaccine efficacy against tuberculosis. Although delayed clearance of BCG may promote immunity by providing continual antigenic stimulation, expanding the nature of the response through enhanced processing and presentation may be a more effective strategy for host protection. It is clear that a strong immune response is initiated during tuberculosis. However, the nature of the response may be equally as important as the magnitude of response. Our data provide new information to be considered on this front.

The cytokine environment at the site of infection is likely to influence the phagosomal/lysosomal pathway. IFN-γ promotes lysosomal fusion with endosomes and acidification [26]. Via et al. showed that IFN-γ treatment increases the acidification of BCG-containing phagosomes in murine macrophages [23]. In this report, bone marrow-derived BCG-infected macrophages from IL-10 deficient mice exhibited increased acidification of BCG phagosomes suggesting that IL-10 has negative influences on the phagosomal/lysosomal pathway [23]. Recently, we have demonstrated that exogenous treatment of IL-27 decreases phagosomal acidification by inhibiting the protein expression of V-ATPases in latex bead-treated human macrophages [21]. IL-27 is expressed by MTB [17,20] and BCG-infected macrophages (Figure 1A). In addition, treatment with IL-12 and sIL-27R restricted growth of both species in human macrophages (Figure 1B, 17, 19, 20). This suggests that IL-27 may create a favorable intracellular environment for the bacteria. Alternatively, blocking IL-27 promotes host protection.

The major finding in this study is that altering the cytokine environment by supplying IL-12 and neutralizing IL-27 resolves the mycobacterial arrest of phagosome maturation. This involves several steps. V-ATPase reduces the pH to 5.0 in mycobacterial phagosomes (Figure 2 and 4) [41]. Eventually BCG-containing phagosomes become phagolysosomes by acquiring CD63 (Figure 3), most likely through fusion with established acidic lysosomes or acquisition from other vesicles through the golgi or ER. Along with the phagosomal acidification, cathepsin D was processed to the mature form (Figure 5) indicating that this enzyme may be actively involved in mycobacterial degradation in an acidic milieu of phagolysosomes. Mature cathepsin D was associated with BCG phagosomes when macrophages were treated with IL-12 and sIL-27R. Cathepsin D may be recruited to phagosomes as a preform and subsequent acidification through acquisition of V-ATPase leads to generation of mature cathepsin D. Enhanced phagosomal acidification and cathepsin D activity during treatment with IL-12 and neutralization of IL-27 was important for limiting intracellular bacterial growth (Figure 6). Inhibition of V-ATPase by bafilomycin and cathepsin D by pepstatin reversed the inhibition of bacterial growth to the level of untreated macrophages (Figure 6C). This data indicates that supplying IL-12 and neutralizing IL-27 specifically influences the phagosomal/lysosomal pathway. IFN-γ was shown to be an important immunological mediator. Neutralizing IFN-γ reversed the enhancement of phagosomal acidification and cathepsin D activity that were responsible for control of mycobacterial growth (Figure 7). This was consistent with reduced V-ATPase and CD63 expression levels along with prevention of cathepsin D maturation (Figure 7). IFN-γ is known to enhance phagosomal acidification and lysosomal fusion [26]. IFN-γ treatment enhanced the fusion of MTB-containing phagosomes with lysosomes in THP-1 cells, and these lysosomes originated from autophagy-stimulated autophagosomes [42]. Since IFN-γ plays a major role in mediating phagosomal acidification during treatment with IL-12 and sIL-27R, an autophagic mechanism may be involved. This is currently under investigation. IFN-γ activates STAT-1 activity to induce anti-microbial mechanisms in macrophages [12]. We found that STAT-1 inhibition reverses lysosomal acidification and association with BCG during treatment with IL-12 and sIL-27R (Figure 8). It is important to point out that although our experiments suggest that STAT-1 associated with IFN-γ signaling promotes lysosomal acidification and association with BCG, our approach using chemical inhibitors does not distinguish STAT-1 induced by signaling through the IFN-γ receptor from that through the IL-27 receptor. In contrast to the positive action of IFN-γ and STAT-1, IL-27 signals through STAT-3 to oppose lysosomal acidification and fusion with BCG. This was demonstrated by the observation that STAT-3 inhibition in the absence of additional influences on the cytokine environment allowed for lysosomal acidification and association with BCG.

Anti-inflammatory cytokines may strongly influence the intracellular fate of mycobacteria in human macrophages. Previous reports show that anti-inflammatory cytokines, IL-4 and TGF-β are increased in MTB-infected individuals [8]. Another anti-inflammatory cytokine, IL-10 is also involved in preventing acidification of mycobacteria-containing phagosomes [23]. Consistent with this observation, blocking IL-10 signaling following BCG vaccination enhanced protective Th1 and Th17 responses [43]. Since IL-10 also signals through STAT-3, IL-27 and IL-10 may operate similarly to oppose lysosomal acidification and protective immunity. Thus, regulating the cytokine environment to establish an antibacterial state in macrophages will not only be beneficial during infection but may also enhance vaccine-induced responses.

## Conclusions

**Figure 9:**
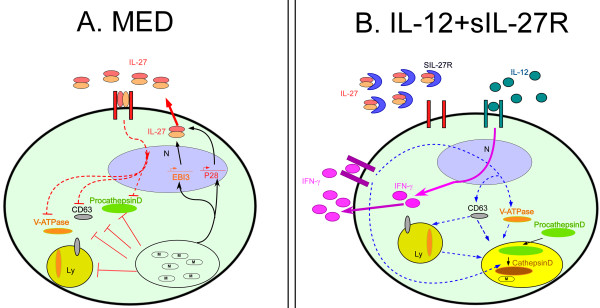
**The effects of IL-12 and IL-27 on human macrophages during mycobacterial infection. (A)** Infected macrophages express p28 and EBI3 resulting in secretion of IL-27. IL-27 downregulates the expression of V-ATPase and negatively regulates other mediators of the phagosomal/lysosomal pathway, such as CD63 and cathepsin D. **(B)** Modulation of the cytokine environment by supplying IL-12 and neutralizing IL-27 enhances the expression of CD63 and V-ATPase resulting in acidification of phagosomes that harbor mycobacteria. CD63 and V-ATPase may be recruited to the phagosome directly. Alternatively, CD63 and V-ATPase may first be recruited to the lysosomes that subsequently fuse with phagosomes. The enhancement of acidification requires IFN-γ and promotes processing of cathepsin D to the mature form.

Mycobacterial infection in human macrophages increases IL-27 production. This blocks mediators of lysosomal acidification and prevented acidification of BCG-containing phagosomes (Figure 9A). Modulating the cytokine environment by supplying IL-12 and neutralizing IL-27 specifically reversed the mycobacterial-mediated phagosomal maturation arrest. This modulation enhanced acidification of the BCG-containing phagosome as well as fusion with lysosomes, consistent with elevated expression of CD63 and V-ATPase. This required IFN-γ production and STAT-1 signaling. Furthermore, the acidic environment enhanced the maturation of cathepsin D and suppressed intracellular growth of bacteria (Figure 9B). This study provides information that should be considered in vaccine development and design of immunotherapeutic approaches to combat TB.

## Methods

### Bacterial strains and growth conditions

*Mycobacterium bovis* Calmette Guérin (BCG) was purchased from ATCC (Manassas, VA). Bacteria were maintained in Middlebrook 7H9 broth supplemented with ADC enrichment media (Albumin, Dextrose, Catalase) at 37°C with 5% CO_2_.

### Staining of *Mycobacterium bovis* BCG

BCG were pre-stained with SYTO-9® or SYTO-61® (100 μM, Molecular Probes, Life Technologies) according to the manufacturer’s instructions. Briefly, 5 × 10^6^ CFUs were pelleted at 2,000 × g for 10 min at room temperature. The supernatant was discarded and the pellet was suspended in SYTO probes at a final volume of 1 mL and passed through a 27-guage needle to disperse the bacteria completely. Following 30 min incubation in the dark, the stained bacteria were centrifuged at 2000 × g for 10 min. The bacterial pellet was suspended in 1 ml of infection medium (DMEM supplemented with 1% human serum, 2 mM glutamine, 25 mM HEPES) and passed through a 27-gauge needle several times. Stained BCG were diluted to a MOI of 10 with infection medium.

### Cell culture

Human buffy coats were purchased from the New York Blood Center (New York, NY). Eligible donors were 16 years of age or older, at least 110 pounds, and in good physical health. The donor samples were anonymous and deidentifed. Peripheral blood mononuclear cells (PBMCs) were isolated from buffy coats by Ficoll gradient centrifugation. Monocytes were then isolated from PBMCs by Optiprep (Sigma-Aldrich) gradient centrifugation as described previously [44]. Monocytes were adhered to plastic 60 mm culture dishes in serum-free DMEM. The media was then replaced with DMEM supplemented with 2 mM glutamine, 25 mM HEPES, 20% fetal bovine serum (FBS), and 10% human serum and incubated at 37°C with 5% CO_2_ for 7 days. Macrophages were removed from the culture dish with PBS that contained 5 mM EDTA and 4 mg/ml lidocaine. The cells were washed with PBS and plated onto new culture dishes in DMEM supplemented with infection medium. These cells are routinely >95% CD14 positive.

### Macrophage infection and enumeration of BCG

Human macrophages were cultivated in 24-well plates (2 × 10^5^/well) and treated with medium alone, IL-12 (5 ng/ml), sIL-27R (10 μg/ml), or their combination for 6 h prior to infection with SYTO-stained BCG (~MOI 10). Infected cultures were incubated 48 h at 37°C with 5% CO_2_. Culture supernatants were removed and macrophages were permeabilized with 1% saponin to release bacteria. Tenfold serial dilutions were plated on Middlebrook 7H10 agar and incubated 10 days at 37°C with 5% CO_2_.

### Analysis of lysosomal acidification and immunolabeling

Human macrophages cultured in 24-well plates were treated as indicated above. In the last hour of infection, culture supernatants were replaced with medium that contained Lysotracker DND-99 Red (Life technologies) (100 nM). The slides were examined using a Zeiss Meta 510 laser confocal microscope with a plan-Apochromat 63× objective lens. A total of 10 fields containing 5–10 macrophages per field were examined in each experiment. The mean fluorescent intensity (MFI) for each macrophage was calculated using Image J software. Each cell from the image was selected and histogram analysis was performed. For immunostaining, mouse monoclonal antibodies for CD63 (sc-5275, Santa Cruz Biotechnology) and V1-ATPase H (sc-166227, santa Cruz Biotechnology) were visualized with anti-mouse-Alexafluor 588-conjugated secondary antibody. Goat polycolonal antibodies for active cathepsin D (sc-6486, Santa cruz) were visualized with anti-goat-Alexafluor 488-conjugated secondary antibody (Life technologies).

### Quantitative bacterial association analysis

To quantify bacterial association with lysosomes, CD63, V-ATPase, or cathepsin D, we employed Pearson’s correlation coefficient analyses. The analyses were performed as follows. During imaging, the microscope pinhole size was set to acquire the same amount of signal for each channel. To minimize the bleed-through effect, the image was scanned sequentially. To avoid saturation, a range indicator for identification of excessive bright and excessive high contrast images was established. This allowed for adjustment using an offset function. Each image was analyzed by image J software. This software produces a number of coefficients for estimating the degree of association. Pearson’s correlation was employed to analyze association between green (BCG) and red (Lysotracker, CD63, V-ATPase) or red (BCG) and green (cathepsin D)) since this analysis considers similarity between shapes [45].

### Quantitative real time PCR

Human macrophages (2×10^5^/well) cultivated in 24-well dishes were treated as indicated. At appropriate time points, media was removed from cultures, the cells were lysed with PureZol® (Bio-Rad), and RNA was isolated according to commercial product protocol. First strand cDNA synthesis was performed using iScript™ cDNA synthesis reagents (Bio-Rad) according to protocol. For IL-27 p28 and EBI3 gene expression analysis, real time cycling of reactions that included cDNA diluted 20-fold from above, gene-specific primer probe sets (Applied Biosystems), iQ™ Supermix (Bio-Rad) was performed in triplicate using iQ5™ cycler (Bio-Rad). GAPDH was used as an internal reference gene.

### Immunoblot analysis

Human macrophages (2 × 10^5^ cells/well) cultivated in 24-well plates were left untreated or treated with the combination of IL-12 (5 ng/ml) and sIL-27R (10 μg/ml) for 6 h prior to infection with BCG (~MOI 10). Infected cultures were incubated for 48 h at 37°C with 5% CO_2_. Briefly, after infection with BCG, 40 μl of PBS supplemented with 1% Tx-100 was applied to each sample and lysates collected by scraping. They were subsequently sonicated and then stored at 4°C. Equal amounts of cell lysates were separated on SDS-PAGE gels and transferred to nitrocellulose by standard technique. Primary antibodies used in this study were mouse monoclonal anti-CD63, V-ATPase H, Cathepsin D antibodies that recognize all forms (sc-374381, Santa Cruz Biotechnology), and rabbit polyclonal anti-actin (Sigma) antibodies. Primary antibodies were revealed with horse radish peroxidase-conjugated anti-mouse or anti-rabbit secondary antibodies. ECL substrate (Amersham Biosciences) was applied to visualize proteins.

### Bafilomycin and pepstatin titration

Bafilomycin and pepstatin were purchased from Sigma. Bafilomycin titration was performed as follows. Macrophages were treated with varying concentrations of bafilomycin (0–1000 nM) for 6 h and then treated with fluorescent-conjugated latex beads for an additional 48 h. The macrophages were examined by confocal microscopy as described earlier in this section. Pepstatin titration was performed as follows. Macrophages were either untreated or treated with varying concentrations (0–100 μM) of pepstatin for 6 h, and then subsequently infected with BCG (MOI 10) for 48 h. Auramine O staining was performed as described previously [46] and fluorescence (λ_ex_ = 420 and λ_em_ = 508 nm) was measured with a Synergy HT Multi-Mode Microplate reader (Biotek, VT).

## Competing interests

The authors declare that they have no competing interests.

## Authors’ contributions

JJ contributed to the design of the experiments and was responsible for the performance of experiments, data analysis, and manuscript preparation. CR conceived the study idea, designed experiments, and contributed equally to the preparation of the manuscript. Both authors read and approve the final manuscript.

## Supplementary Material

Additional file 1: Figure S1 Treatment with IL-12, sIL-27R, or their combination was not toxic to the macrophages. Macrophages were either untreated or treated with IL-12, sIL-27R, or both for 6 h and then subsequently infected with BCG (MOI of 10) for an additional 48 h. Supernatants were collected at 12, 24, and 48 h post-infection and used to assess cell toxicity using a LDH release assay according to the manufacturer’s instruction (Thermo Scientific). The OD 490 nm obtained from lysed macrophages was set to 100%. All other conditions were expressed relative to this value. These data are representative of results from two independent experiments. Click here for file

Additional file 2: Figure S2 Treatment with IL-12, sIL-27R, or their combination did not enhance the formation of lysosomes in the absence of infection. Macrophages were treated with IL-12 and sIL-27R for 48 h. Lysotracker (100 nM) was added at the last hour of incubation. The images shown are representative of three independent experiments. Click here for file

Additional file 3: Figure S3 The combination of IL-12 and sIL-27R induced phagosomal acidification in BCG-infected macrophages. Macrophages were treated with IL-12 and sIL-27R for 6 h prior to infection with SYTO-9® -stained BCG (MOI of 10) for an additional 48 h. Lysotracker (100 nM) was added during the last hour of infection. **(A)** The images shown are representative of three independent experiments (scale bar = 10 μm). **(B)** The mean fluorescent intensity (MFI) was analyzed as described in the Methods section. Click here for file

Additional file 4: Figure S4 Treatment with IL-12 and sIL-27R increased expression of CD63 and V-ATPase in BCG-infected macrophages. Macrophages were treated with IL-12 and sIL-27R for 6 h prior to infection with SYTO-9® -stained BCG (MOI of 10) for an additional 48 h. The cultures were then fixed with 4% PFA, permeabilized, and labeled with anti-CD63 or V-ATPase antibody (red). Representative images from three experiments are shown (scale bar = 10 μm). Click here for file

Additional file 5: Figure S5 The addition of bafilomycin or pepstatin was not toxic to macrophages during infection or treatment with IL-12 and sIL-27R. Macrophages were treated with or without bafilomycin or pepstatin in the presence or absence of IL-12 and SIL-27R for 6 h and then subsequently infected with BCG (MOI of 10) for an additional 48 h. Supernatants were collected at the end of the infection and used to assess cell toxicity using a LDH release assay according to the manufacturer’s instruction (Thermo Scientific). The OD 490 nm obtained from lysed macrophages was set to 100%. All other conditions were expressed relative to this value. These data are combined results from two independent experiments. Click here for file
