# Supplementary figures and images for: IL-12 and IL-27 regulate the phagolysosomal pathway in mycobacteria-infected human macrophages
Source: Cell Commun Signal. 2014 Mar 11;12:16. doi: 10.1186/1478-811X-12-16 (PMC4007735; doi:10.1186/1478-811X-12-16)

Additional file 1: Figure S1.

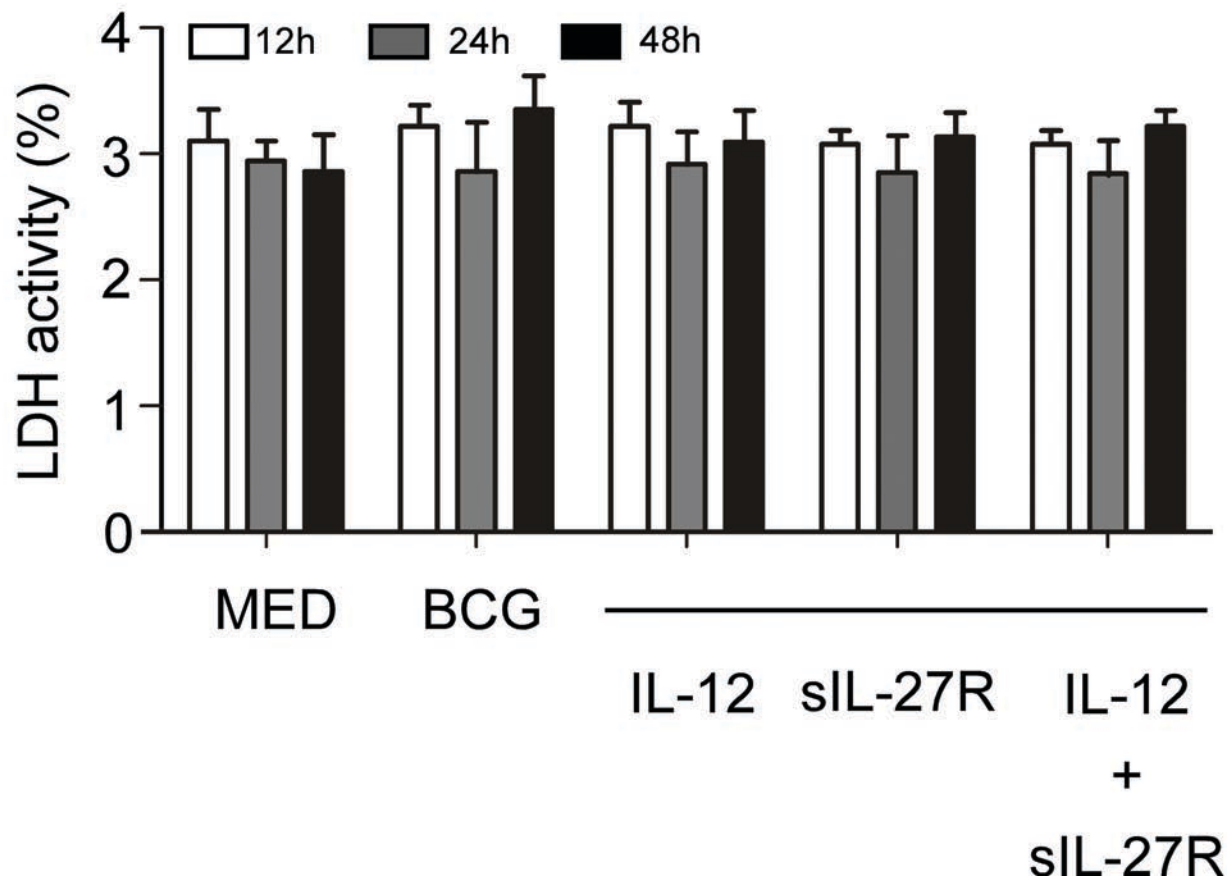

Supplement: Additional file 1: Figure S1 — Treatment with IL-12, sIL-27R, or their combination was not toxic to the macrophages. Macrophages were either untreated or treated with IL-12, sIL-27R, or both for 6 h and then subsequently infected with BCG (MOI of 10) for an additional 48 h. Supernatants were collected at 12, 24, and 48 h post-infection and used to assess cell toxicity using a LDH release assay according to the manufacturer’s instruction (Thermo Scientific). The OD 490 nm obtained from lysed macrophages was set to 100%. All other conditions were expressed relative to this value. These data are representative of results from two independent experiments. [file 1478-811X-12-16-S1.pdf]

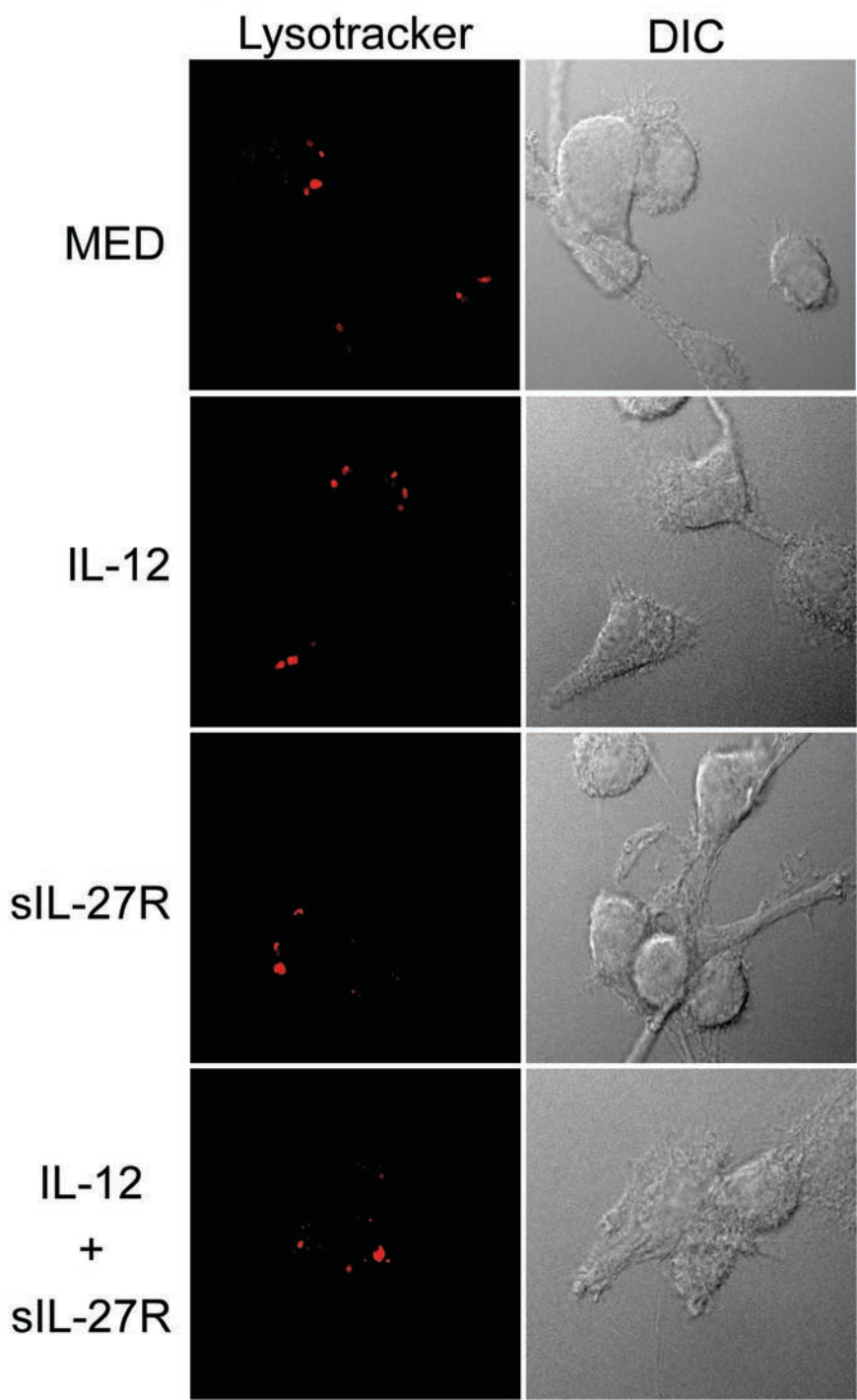

Supplement: Additional file 2: Figure S2 — Treatment with IL-12, sIL-27R, or their combination did not enhance the formation of lysosomes in the absence of infection. Macrophages were treated with IL-12 and sIL-27R for 48 h. Lysotracker (100 nM) was added at the last hour of incubation. The images shown are representative of three independent experiments. [file 1478-811X-12-16-S2.pdf]

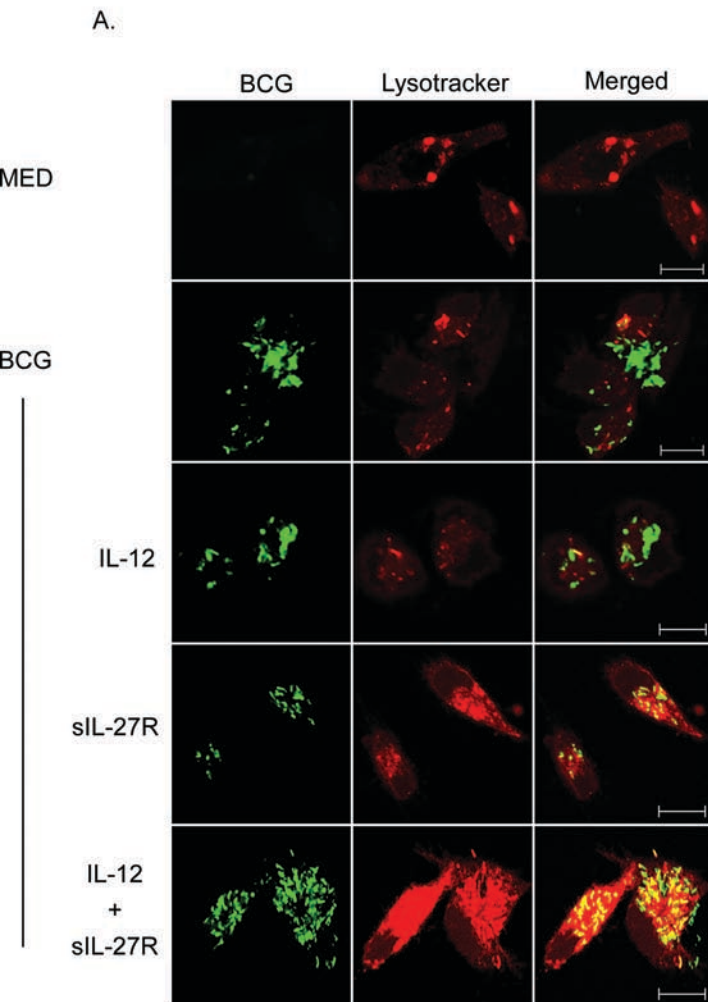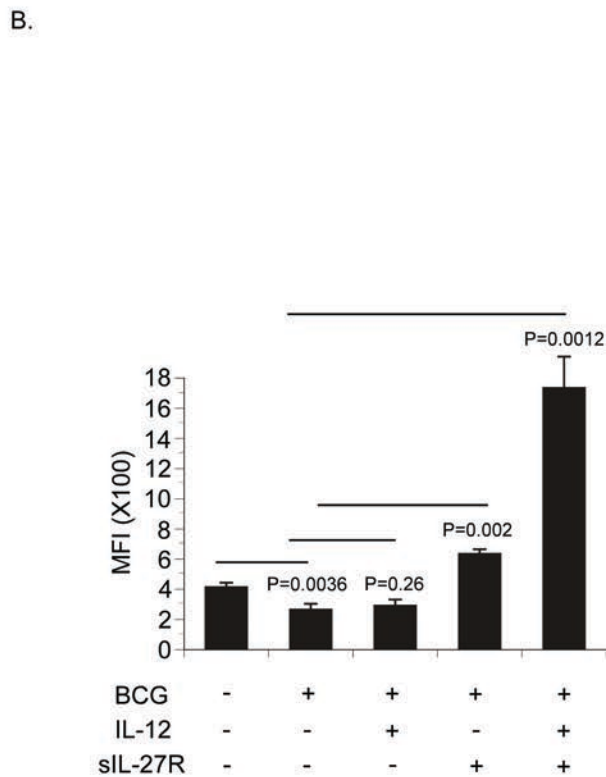

Supplement: Additional file 3: Figure S3 — The combination of IL-12 and sIL-27R induced phagosomal acidification in BCG-infected macrophages. Macrophages were treated with IL-12 and sIL-27R for 6 h prior to infection with SYTO-9® -stained BCG (MOI of 10) for an additional 48 h. Lysotracker (100 nM) was added during the last hour of infection. (A) The images shown are representative of three independent experiments (scale bar = 10 μm). (B) The mean fluorescent intensity (MFI) was analyzed as described in the Methods section. [file 1478-811X-12-16-S3.pdf]

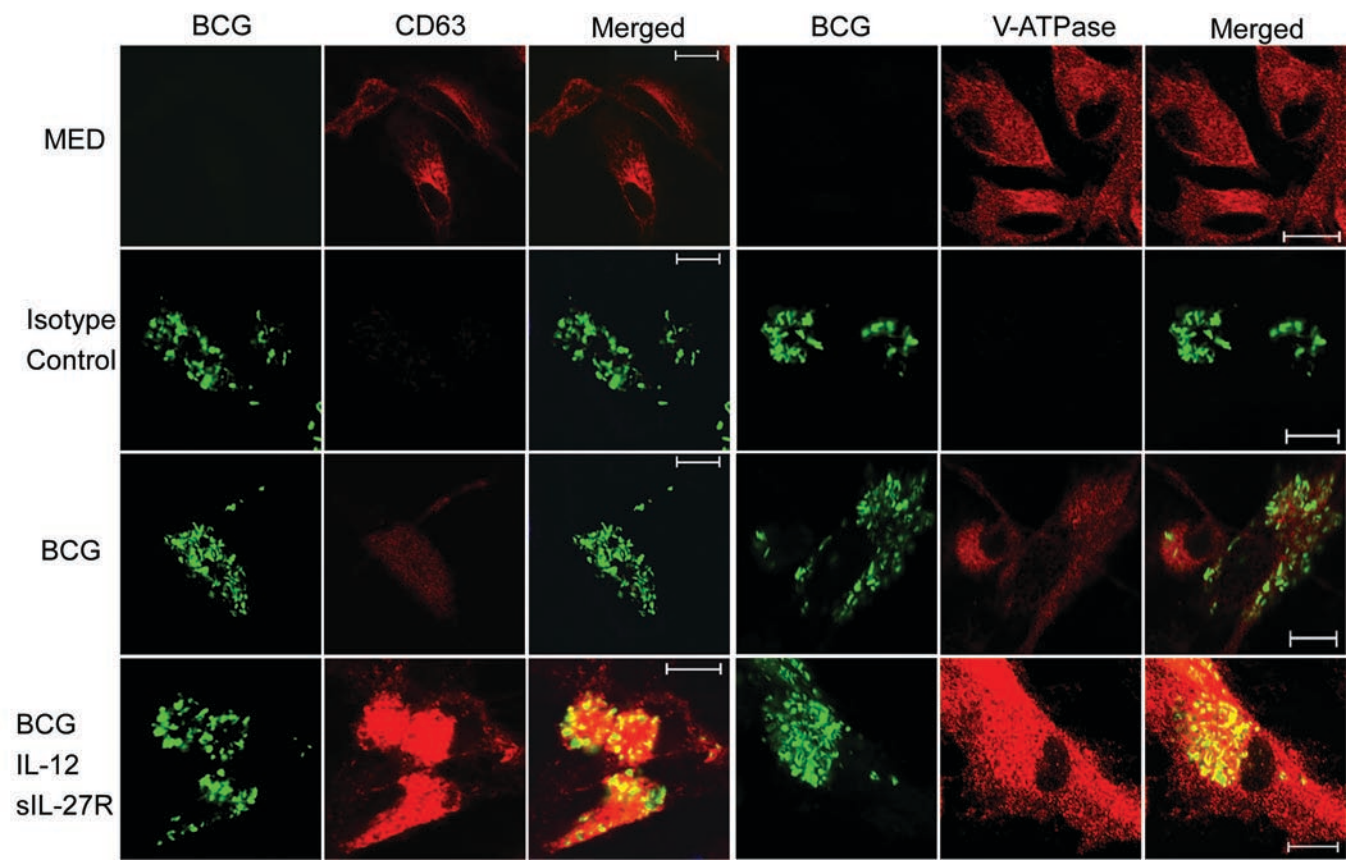

Supplement: Additional file 4: Figure S4 — Treatment with IL-12 and sIL-27R increased expression of CD63 and V-ATPase in BCG-infected macrophages. Macrophages were treated with IL-12 and sIL-27R for 6 h prior to infection with SYTO-9® -stained BCG (MOI of 10) for an additional 48 h. The cultures were then fixed with 4% PFA, permeabilized, and labeled with anti-CD63 or V-ATPase antibody (red). Representative images from three experiments are shown (scale bar = 10 μm). [file 1478-811X-12-16-S4.pdf]

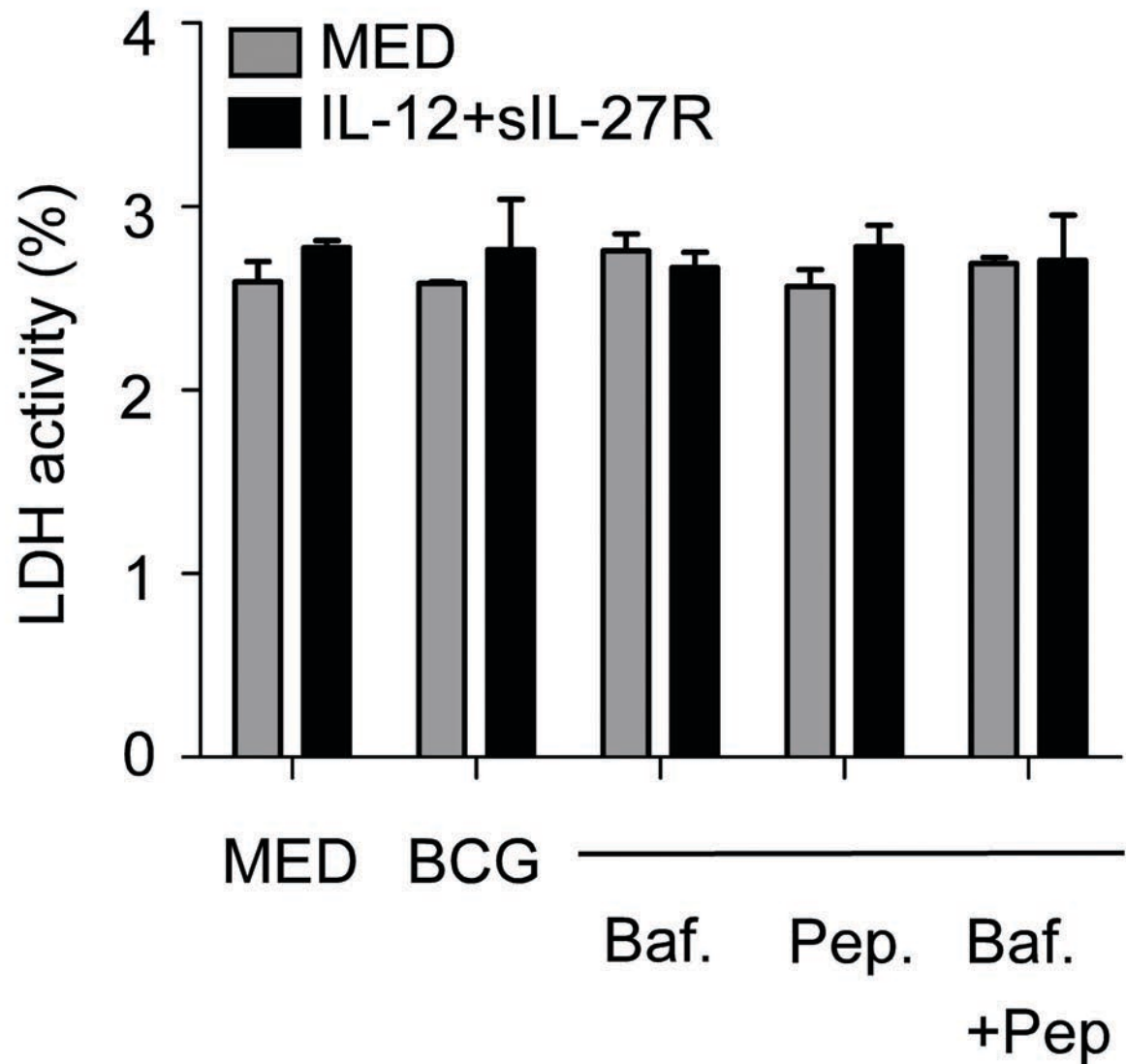

Supplement: Additional file 5: Figure S5 — The addition of bafilomycin or pepstatin was not toxic to macrophages during infection or treatment with IL-12 and sIL-27R. Macrophages were treated with or without bafilomycin or pepstatin in the presence or absence of IL-12 and SIL-27R for 6 h and then subsequently infected with BCG (MOI of 10) for an additional 48 h. Supernatants were collected at the end of the infection and used to assess cell toxicity using a LDH release assay according to the manufacturer’s instruction (Thermo Scientific). The OD 490 nm obtained from lysed macrophages was set to 100%. All other conditions were expressed relative to this value. These data are combined results from two independent experiments. [file 1478-811X-12-16-S5.pdf]
